# Dynamics of the Anatomical Changes That Occur in the Brains of Schoolchildren as They Learn to Read

**DOI:** 10.1371/journal.pone.0081789

**Published:** 2013-12-18

**Authors:** Gregory Simon, Céline Lanoë, Nicolas Poirel, Sandrine Rossi, Amélie Lubin, Arlette Pineau, Olivier Houdé

**Affiliations:** 1 Laboratoire de Pychologie du Développement et de l'Éducation de l'enfant (LaPsyDÉ, Unité CNRS 3521), Université Paris Descartes, Sorbonne Paris Cité, Paris, France; 2 Laboratoire de Psychologie du Développement et de l'Éducation de l'enfant (LaPsyDÉ, Unité CNRS 3521), Université de Caen, Caen, France; 3 Institut Universitaire de France (IUF), Paris, France; University Medical Center (UMC) Utrecht, The Netherlands

## Abstract

Although the functional brain network involved in reading for adults and children is now well documented, a critical lack of knowledge still exists about the structural development of these brain areas. To provide a better overview of the structural dynamics of the brain that sustain reading acquisition, we acquired anatomical MRI brain images from 55 children that were divided into two groups: one prior to the formal learning of reading (n = 33, 5–6 years old) and the second a few years after formal learning (n = 22, 9–10 years old). Reading performances were collected based on the “*Alouette-R*” test, a standardized test for reading text in French. Voxel-based morphometry analysis of gray matter showed that only the right insula volume was different between the two groups. Moreover, the reading group showed that the volumes of the left fusiform gyrus (corresponding to the well-known visual word form area, VWFA), the anterior part of the left inferior occipital gyrus and the left thalamus were significantly modulated by reading performance. This study reinforces the crucial role of the Visual Word Form Area in reading and correlation analyses performed between ROIs volumes suggesting that the VWFA is fully connected with the traditional left-hemispheric language brain network.

## Introduction

Contrarily to oral language, which arose over the course of human evolution, large-scale formal reading instruction in schools appeared no earlier than the end of the 19th century. This strong cultural novelty of civilization raises the following question about the functional and structural (i.e., anatomical) adaptation of schoolchildren's brains when they intensively learn to read between the ages of 5 and 10 years: How does reading acquisition shape children's brain areas and the way that they are connected? In particular, a major gap exists between the reproducible data on the functional network of the brain that is usually found in adults and children during reading tasks [1], [2] and the lack of data regarding the structural development of this network. Formal education might also change the structure of the brain.

According to a functional MRI meta-analysis, reading in adults involves a set of areas that are mainly localized in the left hemisphere [2]. Reading processing activates the posterior middle temporal gyrus, the basal temporal areas and the triangularis part of the inferior frontal gyrus for semantic processing, and the middle/superior temporal gyri, the supramarginalis and opercular part of the inferior frontal gyrus for grapho-phonological conversion. Importantly, a pre-lexical stage of processing occurs in the occipito-temporal junction that is also strongly involved in word recognition and corresponds to the well-known Visual Word Form Area (VWFA). The existence of such an area that is especially devoted to the processing of visual word recognition, was proposed by Dejerine, who described the first case of pure alexia [3] and was intensively studied by Dehaene et al. under the name of VWFA [4]–[6]. This area, which is localized in the left fusiform gyrus at the occipito-temporal junction, was observed to be active early in the development of reading ability. From a structural level, some studies showed Gray Matter (GM) volume modulations in dyslexics, particularly in the temporal regions and in the left fusiform gyrus [7], suggesting that the integrity of this region is crucial for efficient reading. However, the functional role of the VWFA is still under debate, because it was activated in tasks that do not engage visual word form processing [8].

FMRI studies in children suggested a functional network similar to that found in adults soon after reading acquisition [9], [10]. This finding was confirmed by an fMRI meta-analysis showing that children who are approximately 11 years old have a similar set of areas that are recruited during reading tasks as well as other areas such as the thalamus, the SMA, the pre-central gyrus, parietal areas and the right insula [1]. However, even if homologous activation in children and adults were to indicate an early reproducibility of brain regions activation during reading, it does not provide information about the cerebral maturation of these areas, particularly the maturation of gray matter (i.e., GM loss) including synaptic pruning.

In particular situations, such as within guerilla groups in Colombia, children could not acquire reading abilities. In this context, Carreiras et al. [11] described anatomical differences between two groups of children: early- and late-literates deprived of early educational courses. For the late-literate group, these researchers found increased white matter in the splenium of the corpus callosum and greater GM in the bilateral angular, dorsal occipital, middle temporal, left supramarginal and superior temporal gyri. These results suggested that anatomical differences primarily appear in phonological areas and affect grapheme-phoneme conversion. Interestingly, no anatomical differences were observed between early- and late-literates in the VWFA. This was an unexpected result because the VWFA was hypothesized to play a crucial role in the decoding of printed words. This result was also in contradiction with a functional imagery study that showed differences between literate and illiterate adults in the activation of this area [12].

Increasingly, brain-imaging researchers are now focusing on the connectivity between brain regions, as the brain is not a patchwork of regions but rather a large-scale dynamic network in which the different regions are interconnected. According to Fair et al. [13], modularity is a fundamental principle of brain organization. Even if functional connectivity tends to evolve from a local to a distributed network across brain during development, only small changes regarding the modularity of functional brain networks seem to occur between 8 and 25 years, because of a similar “small world” organization for children and adults. However, as mentioned by Fan et al. [14], it is unclear whether structural networks display such real age-related changes. One answer could be found in a study by Zielinski et al. [15] that suggested an evolution of the structural connectivity of speech areas during development; they demonstrated that between 4 years of age and 21 years of age, a progressive, language-related speech and semantic network expanded throughout childhood, with an increasing long-range covariance in early adolescence that progresses throughout the teenage years.

Despite the observed similarity in the functional network involved in reading for adults and young children, an important gap exists between our knowledge of this functional network and the structural development of its brain areas. To investigate the anatomical modulations that occurred during reading acquisition, we conducted a Voxel-Based Morphometry (VBM) study in 5- to 10-year-old children, for which reading abilities were evaluated using the “*Alouette-R*” test [16], a standardized test for reading text in French. We investigated the links between the classical structural reading network regions of interest (ROIs), including the VWFA, and age maturation effects. These analyses were also performed with reading performances for the older group. The aim of the present work is to generate an overview of the structural changes occurring during reading acquisition in the brains of schoolchildren, describing the modulation of GM and structural connectivity. This connectivity was not assessed using DTI but was envisaged as correlations between the volumes (see He et al. [17]) of the different ROIs with the hypothesis that highly connected areas tend to have common developmental pattern and present high correlation coefficients.

## Method

### 2.1. Participants

A total of 55 children recruited from preschools and schools in Caen (Calvados, France) participated in this study; 33 were 5 to 6 years old, and 22 were 9 to 10 years old. The children had no trouble with reading acquisition and had no history of neurological disease. The local ethics committee approved the study (CPP Nord-Ouest III, France). Written consent was obtained from the parents and the children themselves after a detailed discussion and explanation (individual consent for children was adapted as a “smiley” associated with a specific color).

### 2.2. Reading test

Reading proficiency was assessed with the “*Alouette-R*” test, a standardized test for reading text in French [16]. This time-limited text (3 minutes maximum) involves both frequently encountered and very rare words (making them appear as pseudowords to almost any child) and words having a low probability in the sentence context in which they take place. In this study, we assessed speed ((number of words correctly read×180)/reading time) and accuracy (number of words correctly read/number of words read) indexes of reading. This test was provided to children at school a few days after MRI acquisition.

### 2.3. MRI anatomical acquisition

To help the children feel comfortable with the experimenters and experimental material, they first participated individually in a half-hour-long familiarization session at school the day before MRI acquisition in the laboratory. The session consisted of a “statue game”, in which they needed to stay as still as a statue in a plying tunnel imitating the MRI scanner and its technological environment, including the recorded noises of MRI sequence. The day of the MRI acquisition in the laboratory, the same familiarization phase was repeated just prior to the experiment, and the children watched cartoons during the anatomical acquisition inside the magnet.

Three-dimensional (3D) T1-weighted spoiled gradient images (field of view [FOV] = 256 mm, slice thickness = 1.33 mm, 128 slices, matrix size = 192×192 voxels, and duration = 5 min 7 s) were acquired using a 3T MRI scanner (Achieva, Philips Medical System, Netherlands) while the children passively watched a cartoon on an MRI compatible screen to reduce motion in the magnet.

### 2.4. Data analysis

The T1 images were spatially normalized and segmented with SPM5 software© (Wellcome Department of Cognitive Neurology, www.fil.ion.ucl.ac.uk/spm) using a specific template built using the T1 images of the same sample of children. This segmentation created three tissue class images corresponding to gray matter, white matter and cerebrospinal fluid which sum equaling Total Intracranial Volume (TIV). Modulated images were used to perform the statistical analysis and then smoothed (8-mm full-width at half-maximum Gaussian kernel).

Afterwards, two different approaches were used to analyze data. The first approach consisted of a global regression analysis using SPM5. This method was used to assess the cerebral locus of the reading speed index (using as covariate), including a TIV correction. For the second, children reading ROIs from the meta-analysis of Houdé and colleagues [1] were used as a mask to extract the volumes of each ROI in the modulated gray matter images of the 55 children with MATLAB© software (Table 1). Values for each ROI were then analyzed using JMP© software (SAS Company) with a linear least square model including sex, hand preference, age, TIV and the factor of interest (reading speed index). Based on the question being investigated, these analyses were performed on the totality of the children or on a subgroup of them (youngest or oldest). These ROIs included the anterior basal part of the Left Supplementary Motor Area (L SMA), the occipito-temporal junction in the left fusiform gyrus (−44 −52 −18, in an area named Left Visual Word Form Area, L VWFA), the triangularis part of the Left Inferior Frontal Gyrus (L IFG), the posterior part of the Left Middle Temporal Gyrus (L MTG), the bottom anterior part of the Left Precentral Gyrus/opercularis part of Inferior Frontal Gyrus (L PG/IFG), the anterior part of the Right Insula (R Insula), the Left Thalamus (L Thalamus), the Left Inferior Parietal Gyrus (L IPG, at the junction of the inferior and superior parietal gyrus), the posterior part of the Left Inferior Temporal Gyrus (L ITG), the bottom posterior part of the Left Precentral Gyrus (L PG), the Left anterior part of Inferior Occipital Gyrus (L antIOG) and the Left Inferior Occipital Gyrus (L IOG).

**Table 1:** Reading ROIs of the meta-analysis of Houdé et al (2010) [1].

	volume (mm3)	x	y	z
L SMA	1776	−4	10	58
L VWFA	1216	−44	−52	−18
L IFG	1104	−48	32	6
L MTG	728	−52	−42	6
L PG/IFG	600	−44	10	30
R Insula	576	34	24	−2
L Thalamus	408	−10	−14	8
L IPG	272	−30	−58	48
L ITG	96	−56	−50	−12
L PG	64	−40	−4	40
L antIOG	56	−46	−70	−14
L IOG	16	−32	−92	−12

ROIs used to exact gray matter volumes for analysis (L SMA: Left Supplementary Motor Area; L VWFA: Left Visual Word Form Area; L IFG: Left Inferior Frontal Gyrus; L MTG: Left Middle Temporal Gyrus; L PG/IFG: Left Precentral Gyrus/Inferior Frontal Gyrus; R Insula: Right Insula; L Thalamus: Left Thalamus; L IPG: Left Inferior Parietal Gyrus; L ITG: Left Inferior Temporal Gyrus; L PG: Left Precentral Gyrus; L antIOG: Left anterior part of Inferior Occipital Gyrus; L IOG: Left Inferior Occipital Gyrus).

Connectivity was calculated using significant correlations assessed between ROI volumes with JMP© software and then displayed with the BrainNet Viewer software (http://www.nitrc.org/projects/bnv/).

## Results

The VBM technique was used to uncover structural changes that occurred based on age and reading proficiency. T1 images of 55 children were normalized, segmented and quantified for GM volumetry. Global hemispheric volume values, total intracranial volume (TIV = GM+WM+CSF) and GM ROI volumes were extracted from the developmental reading fMRI meta-analysis ROIs of Houdé et al. [1] (Table 1).

### 3.1. The influence of age on the maturation of reading areas

The question being addressed here was whether reading areas were anatomically different between young, non-reading (or beginner-reading) children and older, reading children. Our sample was split into two groups based on age: from 5 to 6 years for non- or beginner-reading children (n = 33) and from 9 to 10 years (n = 22) for reading children. These two groups differed in terms of number of words read during the “*Alouette-R*” test (m = 77.8 words for the 10 children that could decipher words out of the 33 children comprising the non-reader/beginner-reading group and 244.5 words for the older children group, *p*<.0001). No sex differences, manual preference differences or hand preference effects were found between the two groups (Chi-squared test = 1.007; *p* = .32, 2.11; *p* = .15, and 1.30; *p* = .25, respectively). Moreover, there was no difference between the two groups regarding the monthly salary of the parents (*p* = .62) and the weight at birth (*p* = .49).

The effect of age on brain volumetry was assessed by regression analyses using sex (boys *vs.* girls), hand preference (right *vs.* left), age class (5–6 *vs.* 9–10 years) as factors on the global volumetry values (e.g., left hemisphere GM) and reading ROI volumes, which were calculated from the GM images of each child. TIV was also added as factor when it was not itself assessed. This TIV was not significantly different between the two age groups and was not modulated by hand preference. However, as is usually observed in adults, a global sex effect was obtained, with boys presenting a larger TIV than girls (LS Mean: 1382.67 cubic centimeters *vs.* 1277.58 cc, respectively; *p* = .0004). Although global GM volume was decreased with age in the two hemispheres, only the right insula was specifically affected by age (*p* = .0155), suggesting that most of the GM anatomical areas for reading were at least partially mature at the structural level early in development (Table 2). Note that most of these area volumes were also modulated by TIV, with larger TIV values being associated with larger ROI volumes.

**Table 2:** Regression analyses on the volumetry of ROIs in the two groups of children.

	Sex	Hand Preference	Age Class	TIV	model R^2^
	(p values)	(p values)	(p values)	(p values)	
**GLOBAL VALUES**					
Left Hemisphere GM	0.184	0.6197	**0.006**	**<0.0001**	0.74
Right Hemisphere GM	0.1157	0.6806	**0.0062**	**<0.0001**	0.72
Left Hemisphere WM	0.0523	0.9986	**0.0303**	**<0.0001**	0.84
Right Hemisphere WM	0.0864	0.9146	**0.0478**	**<0.0001**	0.84
TIV	**0.0004**	0.4656	0.5228	-	0.24
**READING ROIs**					
L SMA	**0.002**	0.1921	0.8767	**<0.0001**	0.51
L VWFA	**0.0159**	0.8484	0.6249	**<0.0001**	0.51
L IFG	0.8649	0.896	0.1374	**0.0095**	0.18
L MTG	0.3475	0.1496	0.2336	**<0.0001**	0.41
L PG/IFG	0.1311	0.2282	0.0814	**<0.0001**	0.31
R Insula	0.5941	0.1531	**0.0155**	**<0.0001**	0.48
L Thalamus	0.2808	0.1523	0.3473	0.0642	0.17
L IPG	0.1114	0.4182	0.1806	**<0.0001**	0.5
L ITG	0.4166	0.3185	0.5694	0.1533	0.1
L PG	0.2307	0.3344	0.8869	0.2082	0.05
L antIOG	0.1794	0.1088	0.8169	**0.0123**	0.27
L IOG	0.4763	0.4373	0.9576	0.0918	0.12

^2^ value of the complete model of the regression. Regression analyzes were performed on volume extracted from the gray matter (GM) and white matter (WM) hemispheres (without cerebellum) and the different ROIs including factor sex (boys vs girls), hand preference (right vs left), age class (corresponding to young vs older children group) and the TIV. The values in the table represent p values except for last column that correspond to R

Interestingly, the left SMA and left fusiform ROIs were modulated by sex in different ways (*p* = .002; *p* = .0159, respectively); in the former, girls had largest volumes, even after the data were adjusted for TIV, age or hand preference (LS Mean: .794 cc *vs.* .712 cc), whereas the opposite pattern was observed in the fusiform ROI (LS Mean: .699 cc *vs.* .745 cc).

### 3.2. Expertise effects on anatomical areas for reading

To investigate the effect of reading expertise while avoiding the confounding effects of age, this analysis focused only on the older group's results (n = 22). For this sub-group of schoolchildren, there was no correlation between age and reading speed index (r^2^ = .06; *p* = .255). Regression analyses were performed with sex, hand preference, age (in months), TIV and the speed index or accuracy index (included separately in the analysis) the of “*Alouette-R*” reading test as factors.

Among the set of ROIs, the volumes of the VWFA (*p* = .045), the left thalamus (*p* = .0082) and the anterior part of the left inferior occipital gyrus (L antIOG) (*p* = .0032) were significantly modulated by the reading speed index (Table 3). With regard to the VWFA and the L antIOG ROIs, the faster the children decoded words, the weaker its volume was, independent of the decrease due to age modulation. For the thalamus, the correlation with the speed index differed, with high scores being associated with larger GM volumes.

**Table 3:** Effects of the reading speed index on the anatomical ROIs for older children.

	Sex (p values)	Hand Preference(p values)	Age (in months) (p values)	TIV (p values)	Speed index (p values)	model R^2^
**GLOBAL VALUES**						
Left Hemisphere GM	**0.0005**	0.4591	**0.0133**	**<0.0001**	0.2713	0.89
Right Hemisphere GM	**0.0003**	0.5507	**0.0469**	**<0.0001**	0.3111	0.86
Left Hemisphere WM	0.2236	0.9005	0.2425	**<0.0001**	0.538	0.85
Right Hemisphere WM	0.357	0.9294	0.0715	**<0.0001**	0.4985	0.87
TIV	**0.0011**	0.4773	0.8096	-	0.6696	0.51
**READING ROIs**						
L SMA	**0.0026**	0.8235	0.5407	**<0.0001**	0.9544	0.68
L VWFA	0.2046	0.0843	**0.0221**	**0.0006**	**0.045**	0.83
L IFG	0.5234	0.8192	0.2733	0.2943	0.7663	0.15
L MTG	0.9016	0.2445	0.303	**0.04**	0.2908	0.49
L PG/IFG	0.3132	0.4862	0.6168	**0.0489**	0.6885	0.31
R Insula	**0.0398**	0.8149	**0.0384**	**0.0007**	0.3198	0.64
L Thalamus	0.7922	0.8533	0.3595	0.4133	**0.0082**	0.42
L IPG	0.105	0.444	0.4233	**0.0003**	0.1459	0.64
L ITG	0.5674	0.6053	0.6998	0.0778	0.3653	0.27
L PG	0.5995	0.4781	0.9105	0.6666	0.7467	0.08
L antIOG	0.6385	**0.0054**	0.3394	**0.0031**	**0.0032**	0.69
L IOG	0.2872	0.3726	0.6949	0.7663	0.5484	0.17

^2^ value of the complete model of the regression. The volumes of the VWFA, the left thalamus and the anterior part of the left inferior occipital gyrus (L antIOG) were significantly modulated by the reading speed index. The values in the table represent p factor values except for last column that correspond to R

None of the ROIs were modulated by the global reading accuracy index or by the number of errors during the text reading of the “*Alouette-R*” test, suggesting that the maturation of this network – at least at the anatomical level – has a greater influence over the general capacity and automation of reading than its accuracy.

**Figure 1:**
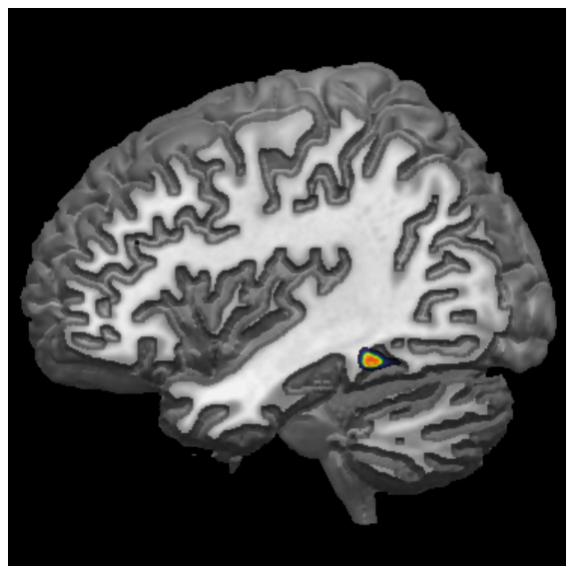
Negative covariation with reading speed index. VBM analysis revealed only one area that correlated negatively with the reading speed score: the VWFA (MNI coordinates −43, −43, −15, *p* value <.001 uncorrected).

To complete the ROI approach, a whole-brain analysis was performed with the reading speed index used as a covariate for a global GM analysis in a SPM5 regression model. The subgroup of 22 schoolchildren presented only one cluster with a negative correlation between volume and the reading speed score located in the left fusiform gyrus (local maximum of MNI coordinates −43, −43, −15; for a *p* value <.001 uncorrected; k = 92 voxels) corresponding to the VWFA (Figure 1), reinforcing the crucial role of this area in reading.

### 3.3. ROI anatomical connectivity

The question addressed here was whether the volume of an ROI depends on the volume of another. If such a relation exists, we hypothesize that links exist between these areas due to maturational and/or expertise factor interactions.

**Figure 2:**
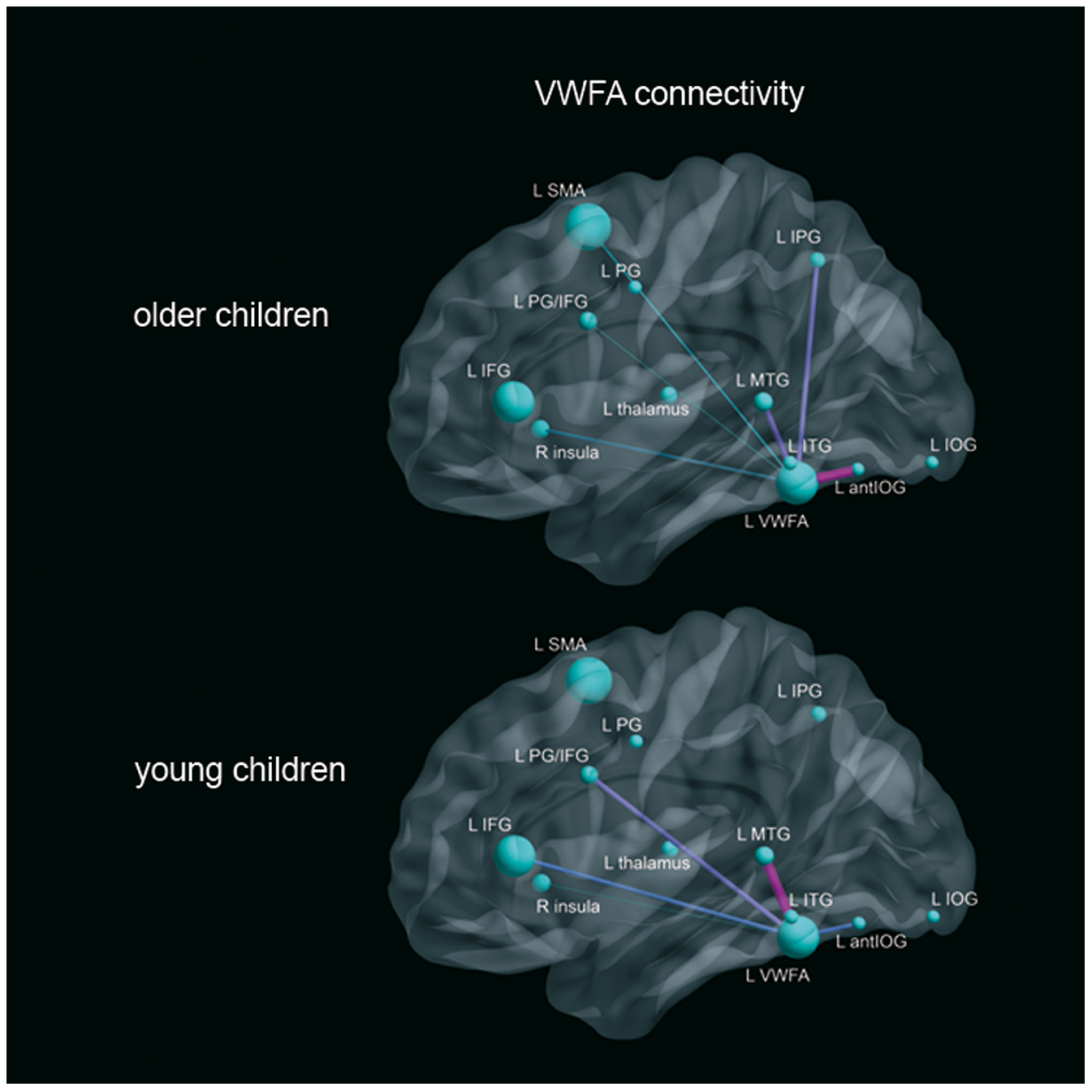
VWFA positive structural correlations in the two groups of children. Volume correlations of the VWFA reading ROIs. Only significant correlations were indicated. The color and the thickness of the lines between the ROIs corresponds to the relative size of the correlation coefficient, warm colors correpond to larger correlations.

In our sample of 22 older children, all significant correlations were positives (Table 4). The larger correlations were obtained with the right insula and the left SMA (r^2^ = .73). A larger number of significant correlations was obtained for the VWFA, which was connected, in order of importance, to the left anterior occipital, left middle temporal, left inferior parietal, right insula, left SMA and left precentral areas (Figure 2).

**Table 4:** Correlations between ROI volumes according to the group of children.

	L SMA	L VWFA	L IFG	L MTG	L PG/IFG	R Insula	L Thalamus	L IPG	L ITG	L PG	L antIOG	L IOG
**L SMA**	-/-	.48/-	.48/-	-/.37	.54/.65	.73/.50	-/-	.65/.56	-/-	-/-	-/-	-/-
**L VWFA**	.48/-	-/-	-/.44	.55/.55	.46/.46	.50/.35	-/-	.54/-	-/-	-/-	.61/.44	-/-
**L IFG**	.48/-	-/.44	-/-	-/.42	-/.44	.63/.50	-/-	-/-	-/-	-/-	-/-	-/-
**L MTG**	-/.37	.55/.55	-/.42	-/-	-/-	-/.62	-/.42	-/-	.51/.40	-/-	.58/.49	-/-
**L PG/IFG**	.54/.65	.46/.46	-/.44	-/-	-/-	.55/.48	-/-	.51/.36	-/-	-/-	-/-	-/-
**R Insula**	.73/.50	.50/.35	.63/.5	-/.62	.55/.48	-/-	-/.40	.70/.42	-/.39	-/-.38	-/.37	-/.37
**L Thalamus**	-/-	-/-	-/-	-/.42	-/-	-/.40	-/-	-/-	-/-	-/-	-/-	-/-
**L IPG**	.65/.56	.54/-	-/-	-/-	.51/.36	.70/.42	-/-	-/-	.44/-	-/-	-/-	-/-
**L ITG**	-/-	-/-	-/-	.51/.40	-/-	-/.39	-/-	.44/-	-/-	-/-	-/.39	-/-
**L PG**	-/-	-/-	-/-	-/-	-/-	-/-.38	-/-	-/-	-/-	-/-	-/-	-/-.42
**L antIOG**	-/-	.61/.44	-/-	.58/.49	-/-	-/.37	-/-	-/-	-/.39	-/-	-/-	-/-
**L IOG**	-/-	-/-	-/-	-/-	-/-	-/.37	-/-	-/-	-/-	-/-.42	-/-	-/-

Between the ROIs, only significant correlation coefficients were reported for older/young children.

Note that the younger group (n = 33) demonstrated quite different correlation profiles. Initially, we obtained negative correlations for the left precentral ROI with the more posterior occipital ROI (r^2^ = −.42) and the right insula ROI (r^2^ = −.38). The largest correlation was observed for the precentral and SMA ROIs (r^2^ = .65), and the region of the right insula was the one that presented the largest numbers of significant correlations, whose value correlated with all other ROI volumes.

These analyses indicated important differences in young and older children between the connectivity of VWFA ROIs. In the older group, the highest correlation was obtained between the L antIOG and the VWFA (r^2^ = .61). This correlation was more spread in the pre-literate, younger group of children (r^2^ = .44). However, this last result should be replicated with a larger sample size because Fisher transformation demonstrates that this difference was not significant (z_1_–z_2_ = 0.24; confidence interval: −0.34/0.81). In the younger group, the best correlation score with the VWFA was observed for the left middle temporal gyrus (r^2^ = .55), which was a value that was similar than those found for the older group. Interestingly, a significant link between the VWFA and the left IPG (r^2^ = .54) was found only in the older group.

## Discussion

The aim of the present study was to investigate the structural maturation of brain regions that are involved in reading in schoolchildren. Our results were in agreement with the early maturity of the reading brain network suggested in other studies [9], [10]. Indeed, we found no age effect between young, non-reading/beginner-reading children and older reading children for reading ROIs (except for the right insula) when data were corrected for TIV differences. Therefore, independent of age modulation *per se*, our results revealed that the development of reading efficiency in schoolchildren was underlined by structural modulation within the left occipital, VWFA and thalamus regions. These very different and important brain areas were often activated during reading in fMRI studies [6], [12], [18]. Here, we further demonstrate that reading skill modulates the structural evolution of these regions and their connectivity in a different manner.

### 4.1. The left fusiform gyrus is a crucial area in reading development

Numerous fMRI studies have shown the crucial role for the left fusiform gyrus, and more precisely the region corresponding to the VWFA, in adult reading [6]. Moreover, the fMRI study of Gaillard et al. [10] suggested that the functional brain network for reading was very similar in adults and in 7-year-old children (see also [9]). Our structure-based results agreed with this hypothesis. Indeed, we found that only the right insula was modulated by age between our two groups of 6- and 10-year-old children when TIV was included in the statistical model, suggesting that most of the GM anatomical regions involved in reading are at least partially mature early in development.

Nevertheless, even if the volume of the VWFA GM in young and older children was not significantly modulated by age, we found an effect of reading skill on the volumetry of this area for the older group. In our study, the better performances were associated with smaller GM volume, whereas the index of reading skill was not correlated with the age of the children. Even if some studies evidenced positive correlations between GM and behavioral scores (see thereafter for further details), it was also previously found in adults – as in the present results in children - that a smaller fusiform gyrus GM volume could be related to better phonological awareness scores [19]. The volume of the fusiform gyrus GM was also greater for normal compared to adults dyslexic readers. This result seems contradictory because better performances are associated with a smaller GM volume in normal populations, whereas dyslexics, who present reading impairment, had less GM than the normal-reading population (see also [20]). It may that the GM volume deficit in left fusiform and other temporal regions observed in dyslexia would be present even before reading acquisition [21]. The results from dyslexia studies and their inferences about the “typical” scheme of reading development must be interpreted with caution. Even if our study did not focus particularly on sex differences, we found results such an effect on the VWFA, in which boys had larger GM volumes than girls for the equivalent TIV. More investigations should be performed to explain this modulation, but such a sex effect could most likely be used as a clue to explain sex proportion differences that exist in developmental alexia [22].

Our study demonstrated a relation between the diminution of GM volume and better reading skills in children. According to the fMRI literature, it appears that the development of a reading system (from 7 to 18 years old) consists of an increase in the occipito-temporal region and a decrease in the frontal region activities with age [23]. In adults, an fMRI study suggested a positive correlation between the activity of the VWFA and reading performance within unschooled adults [12]. This apparent discrepancy could be explained either by the tasks used in this study, by the absence of a link between anatomical and functional results, or more likely, by the fact that the link between functional activity and structural maturation/modulation is more complex than it appears. Note that some fMRI studies failed to show a positive correlation between reading performance and the VWFA [24]. It may be that the link between functional and structural modulation rests upon several processes (this point will be developed in the last section of the discussion).

### 4.2. Reading skills and other regions

In addition to VWFA, two other reading ROI volumes were significantly modulated by reading skill and more precisely by the reading speed index: the left thalamus and the L antIOG ROI. The finding of a left occipital area whose volume correlates with the reading speed index in a similar manner as the VWFA is consistent because of spatial proximity. This occipital ROI (MNI coordinate −46, −70, −14) was near to the one shown in the study of Dehaene et al. [12], which showed an increase in its activity with literacy but for all visual stimuli with high contrast, not just letters. This area (as with most of the occipital areas) was more highly activated during mirror reading than normal reading [25], and the left inferior occipital gyrus was also more highly activated during the reading of Chinese vs. meaningless figures [26]. Therefore, this area seems important in the reading process for low-level visual processing, perhaps by improving letter discrimination that conveys bottom-up information to the VWFA.

We also obtained an effect of reading performance on the GM volume of the left thalamus. Numerous fMRI studies have shown activity of this sub-cortical area during a reading task [18], [27]. This region is most likely not an area of reading *per se* but a relay for passing sensory information to cortical regions. For instance, in a resting-state functional connectivity study [28], Koyama et al. showed that reading in children may rely more on the increased connectivity between the cortical intraparietal sulcus and the subcortical thalamus attention region compared to adults. Our results also suggest such a link, as we observed an effect in children of reading performance on this information crossroad involving the thalamus. Because reading is less automated in children than in adults, it likely requires more cognitive effort and attention, which leads to the more intensive involvement of the thalamus. However, contrary to what we observed for the VWFA and the inferior occipital areas, a positive correlation of thalamic GM volume and the reading speed index was discovered; this subcortical structure may be more mature in children than other cortical ROIs (see the last section of the discussion for further explanation).

### 4.3. Structural Connectivity

Connectivity was also used in the present work to further study the reading network in the brains of children. The question addressed here was whether the GM volume of an ROI depends on the volume of another. If such a relation exists, then we can hypothesize that links exist between areas due to maturational or expertise factors. At a global level, our results suggested larger correlation values for older children that could correspond to a more mature anatomical network in that group or the presence of more inter-individual differences in the younger group.

In the older group of children, only positive correlations were obtained, and a larger number of significant correlations was found for the VWFA, which was “connected”, in order of importance, to the anterior L antIOG ROI, the left middle temporal, the left inferior parietal, the right insula, the left SMA, and the left pre-central regions. This is in agreement with the study of van der Mark et al. [29], who demonstrated with fMRI that, in the occipito-temporal region, only the VWFA was functionally connected with the remote brain areas of the traditional left-hemispheric language network.

As was found in the study of Levy et al. [30], using functional connectivity during word, pseudo-word and non-word reading, we obtained a positive correlation between the L antIOG region and the VWFA. These two areas are most likely tuned to quickly recognize letters and words. This reinforces the hypothesis that this occipital area transmits bottom-up information to the VWFA. We also observed differences between our young and older groups. Indeed, in skilled-reading children, the volume of these two areas was modulated by reading efficiency, and the volume of the L antIOG ROI presented the larger correlation coefficient with the VWFA. A different result was obtained in group of younger children, for which the highest correlation with the VWFA was elicited by the MTG. These results support that, under pressure from expertise, the GM of L antIOG and VWFA tends to evolve in a similar way and that this evolution could represent the development of anatomical and therefore functional interactions between them.

Using fMRI rest connectivity, it was also shown that the VWFA was more connected to dorsal attention regions than reading areas *per se* [31]. Previous studies suggested that the VWFA was a more general visual processor that was used in varied visual tasks, including reading tasks but was not specific to printed words. However, our results showed that reading performance was dependent on the volume of this region. Moreover, we obtained numerous structural connectivity indices between the VWFA and regions typically involved in reading. Interestingly, our results also showed a correlation between the VWFA and the left inferior parietal seed that was obtained only in the reading child group, suggesting that learning to read develops anatomical connectivity between orthographic and phonological/semantic areas [2], [32], [33].

### 4.4. Increase and decrease dynamic of GM in children development

The GM modulation and its interaction with cognitive abilities during lifespan is a very complex process. One could question why reading skills in our study were associated with both the loss and the increase of GM in different brain areas. According to Shaw et al. [34], GM development is not linear. In their longitudinal study, they found a predominantly negative correlation between IQ and cortical thickness in an early childhood group (from 3.8 to 8.4 years); this correlation was in contrast with later, positive correlations that peaked in late childhood (from 8.6 to 11.7 years) and that were present in an attenuated form in adolescent and early adult groups. This study supports the hypothesis that GM development is a dynamic process that evolves with age, but it does not explain why, at the same age, several brain regions may have inverse patterns of modulation, as was the case in our study.

In adults, Draganski et al. [35] showed regional GM volume increases after 3 months of juggling training (see also [36]). More similar to our reading task, Ilg et al. [25] also demonstrated that practice of mirror reading daily for 15 min over 2 weeks was sufficient to increase performance, which was correlated to a decrease in fMRI activation in the right superior parietal cortex and an increase in activation in the right dorsal occipital cortex. Moreover, this longitudinal study demonstrated an increase in the GM in the right dorsolateral occipital cortex that corresponded to the peak of mirror reading-specific activation and suggested that short-term practice modulates regional GM variation. Their findings supported the hypothesis that learning-induced cortical plasticity is also reflected at a structural level and that training could produce an increase in GM. This GM increase could also occur in children. One study showed such a modification of GM volume localized in the left anterior fusiform gyrus in 9-year-old dyslexic children after reading skill training [37].

The increase and loss of GM might be associated with two different processes in development that require intensive study in future experiments. In our study, we found a negative correlation between the reading speed index for the fusiform and occipital ROIs and a positive correlation for the thalamus in our group of 9- to 10-year-old children. Interestingly, the thalamus is an archaic structure compared to the cortex. Therefore, one could hypothesize that this structure is mature at the age of 10, whereas other areas, such as the VWFA, are not. The GM decrease was likely due to a synaptic pruning process that could represent a maturation stage that occurs until a critical period. This pruning would explain the acute plasticity of children's brains that occurs at different times in different regions. After this initial pruning, we can hypothesize that the GM increase can compensate partially for new learning, but subsequent to the pruning phase, these areas are most likely less flexible in terms of neural synaptic modulation. Based on this assumption, our results suggest that, at the age of 10, the VWFA is not totally mature and is always in the pruning stage. As a consequence, the absence of an age effect (when the data were corrected by TIV) on the VWFA shown between our 2 groups of children (younger *vs.* older) did not indicate that the VWFA was mature early. Rather, it suggests that the structural maturation of this structure occurs late.

## Conclusions

This study reinforces the crucial role of the fusiform gyrus, and more especially the Visual Word Form Area in reading. The present results also reveal crucial information regarding reading development, showing that independently of their age, gray matter of the VWFA is modulated by the reading performances of the children. Moreover, the volume of the VWFA is correlated with other reading brain ROIs, suggesting that the VWFA is fully connected with the traditional left-hemispheric language brain network.

## References

[1] Houdé O, Rossi S, Lubin A, Joliot M (2010) Mapping numerical processing, reading, and executive functions in the developing brain: an fMRI meta-analysis of 52 studies including 842 children. Dev Sci 13: 876–885 10.1111/j.1467-7687.2009.00938.x 20977558

[2] Jobard G, Crivello F, Tzourio-Mazoyer N (2003) Evaluation of the dual route theory of reading: a metanalysis of 35 neuroimaging studies. Neuroimage 20: 693–712 10.1016/S1053-8119(03)00343-4 14568445

[3] Dejerine J (1892) Contribution a l'étude anatomo-pathologique et clinique des différentes variétés de cécité verbale. Mémoires de la Société de Biologie 4: 61–90.

[4] Cohen L, Dehaene S, Naccache L, Lehéricy S, Dehaene-Lambertz G, et al (2000) The visual word form area: spatial and temporal characterization of an initial stage of reading in normal subjects and posterior split-brain patients. Brain 123 (Pt 2) 291–307. 10648437 10.1093/brain/123.2.291

[5] Epelbaum S, Pinel P, Gaillard R, Delmaire C, Perrin M, et al (2008) Pure alexia as a disconnection syndrome: new diffusion imaging evidence for an old concept. Cortex 44: 962–974 10.1016/j.cortex.2008.05.003 18586235

[6] Dehaene S, Cohen L (2011) The unique role of the visual word form area in reading. Trends Cogn Sci (Regul Ed) 15: 254–262 10.1016/j.tics.2011.04.003 21592844

[7] Brambati SM, Termine C, Ruffino M, Stella G, Fazio F, et al (2004) Regional reductions of gray matter volume in familial dyslexia. Neurology 63: 742–745. 15326259 10.1212/01.wnl.0000134673.95020.ee

[8] Price CJ, Devlin JT (2003) The myth of the visual word form area. Neuroimage 19: 473–481. 12880781 10.1016/s1053-8119(03)00084-3

[9] Church JA, Coalson RS, Lugar HM, Petersen SE, Schlaggar BL (2008) A developmental fMRI study of reading and repetition reveals changes in phonological and visual mechanisms over age. Cereb Cortex 18: 2054–2065 10.1093/cercor/bhm228 18245043 PMC2517103

[10] Gaillard WD, Balsamo LM, Ibrahim Z, Sachs BC, Xu B (2003) fMRI identifies regional specialization of neural networks for reading in young children. Neurology 60: 94–100. 12525725 10.1212/wnl.60.1.94

[11] Carreiras M, Seghier ML, Baquero S, Estévez A, Lozano A, et al (2009) An anatomical signature for literacy. Nature 461: 983–986 10.1038/nature08461 19829380

[12] Dehaene S, Pegado F, Braga LW, Ventura P, Nunes Filho G, et al (2010) How learning to read changes the cortical networks for vision and language. Science 330: 1359–1364 10.1126/science.1194140 21071632

[13] Fair DA, Cohen AL, Power JD, Dosenbach NUF, Church JA, et al (2009) Functional brain networks develop from a “local to distributed” organization. PLoS Comput Biol 5: e1000381 10.1371/journal.pcbi.1000381 19412534 PMC2671306

[14] Fan Y, Shi F, Smith JK, Lin W, Gilmore JH, et al (2011) Brain anatomical networks in early human brain development. Neuroimage 54: 1862–1871 10.1016/j.neuroimage.2010.07.025 20650319 PMC3023885

[15] Zielinski BA, Gennatas ED, Zhou J, Seeley WW (2010) Network-level structural covariance in the developing brain. Proc Natl Acad Sci USA 107: 18191–18196 10.1073/pnas.1003109107 20921389 PMC2964249

[16] Lefavrais P (2005) Alouette-R: test d'analyse de la vitesse de lecture à partir d'un texte.

[17] He Y, Chen ZJ, Evans AC (2007) Small-world anatomical networks in the human brain revealed by cortical thickness from MRI. Cereb Cortex 17: 2407–2419 10.1093/cercor/bhl149 17204824

[18] Fiebach CJ, Friederici AD, Müller K, von Cramon DY (2002) fMRI evidence for dual routes to the mental lexicon in visual word recognition. J Cogn Neurosci 14: 11–23 10.1162/089892902317205285 11798383

[19] Frye RE, Liederman J, Malmberg B, McLean J, Strickland D, et al (2010) Surface area accounts for the relation of gray matter volume to reading-related skills and history of dyslexia. Cereb Cortex 20: 2625–2635 10.1093/cercor/bhq010 20154011 PMC2981021

[20] Hoeft F, Meyler A, Hernandez A, Juel C, Taylor-Hill H, et al (2007) Functional and morphometric brain dissociation between dyslexia and reading ability. Proc Natl Acad Sci USA 104: 4234–4239 10.1073/pnas.0609399104 17360506 PMC1820738

[21] Raschle NM, Chang M, Gaab N (2011) Structural brain alterations associated with dyslexia predate reading onset. Neuroimage 57: 742–749 10.1016/j.neuroimage.2010.09.055 20884362 PMC3499031

[22] Rutter M, Caspi A, Fergusson D, Horwood LJ, Goodman R, et al (2004) Sex differences in developmental reading disability: new findings from 4 epidemiological studies. JAMA 291: 2007–2012 10.1001/jama.291.16.2007 15113820

[23] Shaywitz BA, Skudlarski P, Holahan JM, Marchione KE, Constable RT, et al (2007) Age-related changes in reading systems of dyslexic children. Ann Neurol 61: 363–370 10.1002/ana.21093 17444510

[24] Turkeltaub P, Gareau L, Flowers D, Zeffiro T, Eden G (2003) Development of neural mechanisms for reading. Nature Neuroscience 6: 767–773. 12754516 10.1038/nn1065

[25] Ilg R, Wohlschläger AM, Gaser C, Liebau Y, Dauner R, et al (2008) Gray matter increase induced by practice correlates with task-specific activation: a combined functional and morphometric magnetic resonance imaging study. J Neurosci 28: 4210–4215 10.1523/JNEUROSCI.5722-07.2008 18417700 PMC6670304

[26] Kuo WJ, Yeh TC, Duann JR, Wu YT, Ho LT, et al (2001) A left-lateralized network for reading Chinese words: a 3 T fMRI study. Neuroreport 12: 3997–4001. 11742227 10.1097/00001756-200112210-00029

[27] Rosen HJ, Ojemann JG, Ollinger JM, Petersen SE (2000) Comparison of brain activation during word retrieval done silently and aloud using fMRI. Brain Cogn 42: 201–217 10.1006/brcg.1999.1100 10744920

[28] Koyama MS, Di Martino A, Zuo X-N, Kelly C, Mennes M, et al (2011) Resting-state functional connectivity indexes reading competence in children and adults. J Neurosci 31: 8617–8624 10.1523/JNEUROSCI.4865-10.2011 21653865 PMC3893355

[29] van der Mark S, Klaver P, Bucher K, Maurer U, Schulz E, et al (2011) The left occipitotemporal system in reading: disruption of focal fMRI connectivity to left inferior frontal and inferior parietal language areas in children with dyslexia. Neuroimage 54: 2426–2436 10.1016/j.neuroimage.2010.10.002 20934519

[30] Levy J, Pernet C, Treserras S, Boulanouar K, Aubry F, et al (2009) Testing for the dual-route cascade reading model in the brain: an fMRI effective connectivity account of an efficient reading style. PLoS ONE 4: e6675 10.1371/journal.pone.0006675 19688099 PMC2724737

[31] Vogel AC, Miezin FM, Petersen SE, Schlaggar BL (2011) The Putative Visual Word Form Area Is Functionally Connected to the Dorsal Attention Network. Cereb Cortex Available: http://www.ncbi.nlm.nih.gov/pubmed/21690259. Accessed 2011 Aug 2. 10.1093/cercor/bhr100 PMC3278314 21690259

[32] Vigneau M, Beaucousin V, Hervé PY, Duffau H, Crivello F, et al (2006) Meta-analyzing left hemisphere language areas: phonology, semantics, and sentence processing. Neuroimage 30: 1414–1432 10.1016/j.neuroimage.2005.11.002 16413796

[33] Turkeltaub PE, Eden GF, Jones KM, Zeffiro TA (2002) Meta-analysis of the functional neuroanatomy of single-word reading: method and validation. Neuroimage 16: 765–780. 12169260 10.1006/nimg.2002.1131

[34] Shaw P, Greenstein D, Lerch J, Clasen L, Lenroot R, et al (2006) Intellectual ability and cortical development in children and adolescents. Nature 440: 676–679 10.1038/nature04513 16572172

[35] Draganski B, Gaser C, Busch V, Schuierer G, Bogdahn U, et al (2004) Neuroplasticity: changes in grey matter induced by training. Nature 427: 311–312 10.1038/427311a 14737157

[36] Draganski B, May A (2008) Training-induced structural changes in the adult human brain. Behav Brain Res 192: 137–142 10.1016/j.bbr.2008.02.015 18378330

[37] Krafnick AJ, Flowers DL, Napoliello EM, Eden GF (2011) Gray matter volume changes following reading intervention in dyslexic children. Neuroimage 57: 733–741 10.1016/j.neuroimage.2010.10.062 21029785 PMC3073149

